# Evidence of personality-dependent plasticity in dairy calf movement behaviours derived from automated data collection

**DOI:** 10.1038/s41598-023-44957-z

**Published:** 2023-10-25

**Authors:** Francesca Occhiuto, Jorge A. Vázquez-Diosdado, Andrew J. King, Jasmeet Kaler

**Affiliations:** 1 School of Veterinary Medicine and Science, University of Nottingham, Sutton Bonington Campus, Leicestershire, LE12 5RD UK; 2 Department of Biosciences, Faculty of Science and Engineering, Singleton Park Campus, Swansea University, Swansea, SA2 8PP UK

**Keywords:** Behavioural ecology, Behavioural methods, Animal behaviour

## Abstract

Individual consistency in behaviour, known as animal personality, and behavioural plasticity in response to environmental changes are important factors shaping individual behaviour. Correlations between them, called personality-dependent plasticity, indicate that personality can affect individual reactions to the environment. In farm animals this could impact the response to management changes or stressors but has not yet been investigated. Here we use ultra-wideband location sensors to measure personality and plasticity in the movement of 90 dairy calves for up to 56 days starting in small pair-housing enclosures, and subsequently moved to larger social housings. For the first time calves were shown to differ in personality and plasticity of movement when changing housing. There were significant correlations between personality and plasticity for distance travelled (0.57), meaning that individuals that travelled the furthest in the pair housing increased their movement more in the social groups, and for residence time (− 0.65) as those that stayed in the same area more decreased more with the change in housing, demonstrating personality-dependent plasticity. Additionally, calves conformed to their pen-mate’s behaviour in pairs, but this did not continue in the groups. Therefore, personality, plasticity and social effects impact how farm animals respond to changes and can inform management decisions.

## Introduction

Behavioural differences between individuals of the same population and species, which are stable in time and across different contexts, are defined as animal personality, and are observed in a variety of animal species^1–3^. In contrast, behavioural plasticity is characterised by behavioural change, where individuals adapt their behaviour in response to changes in the environmental conditions and circumstances^4–6^. Individuals within a population can adjust their behaviour in response to environmental stimuli or changes (plasticity) and still show consistent inter-individual differences in behaviour (personality)^5^. Indeed, the extent of variation in behaviours may be affected by personality differences present in the population^5,7^. For example, more exploratory individuals have been observed to show greater plasticity in behaviour compared to less exploratory individuals in great tits^8^ and bolder individuals were less plastic than shy individuals in sticklebacks^9^. Correlation between personality and plasticity may therefore be widespread, but the causes and consequences of these correlations are not well understood^10,11^.

Quantifying and understanding the consequences of correlations between personality and plasticity in animals requires repeated measurements of behaviour taken over an environmental gradient or in different contexts^12^. This can be difficult to achieve with traditional behavioural observation methods in the laboratory or in the wild^13,14^. With the advent of modern sensor and tracking technologies such data are, however, becoming more accessible^10,15–17^, especially since the movement behaviours easily captured by these technologies directly relate to established personality constructs of “exploration” and “activity”^16,18,19^. An excellent example is the use of precision technology applied to farm animals. These technologies provide repeated and detailed behavioural data that offer the opportunity to advance our understanding of personality and plasticity, whilst improving farm animal management protocols by focussing on individuals’ requirements^20^. Indeed, location sensors have been used to show evidence of personality types regarding activity and exploration in dairy calves^17^, but the effects of environmental changes and any individual differences in behavioural plasticity have not been investigated.

Here, we use automated data collection using ultra-wideband sensors^21^ to investigate personality in dairy calf movement behaviours and the plasticity in these movement behaviours when individuals experience changes to their physical and social environments because of routine management regimes^22^. In the UK, dairy calves are often housed alone or in pairs at birth and then moved to larger social groups in different housings as they grow older^22^. This system therefore presents an ideal set-up for studying the effects of changing environment (housing and social grouping) on farm animal personality and plasticity. Social animals are known to adjust their behaviour to other individuals in the group, a phenomenon known as behavioural synchronicity^23^, as an anti-predator strategy and to increase group cohesiveness^24,25^. However, it is not known whether such social dynamics can lead to long lasting effects on individual personality. In addition, an understanding of how individual characteristics such as personality traits and differences in behavioural plasticity might affect an animal’s response to management interventions could result in more effective and tailored management decisions, with health and welfare benefits^20^. Indeed, recent studies have attempted to use behavioural indicators such as reduced activity to detect disease in cattle^26,27^. However only using group averages—when we know that animals vary in their personality—may result in incorrect detection of disease, limiting the effectiveness of any intervention.

We recorded the movement behaviours of 90 dairy calves for up to 56 days, starting as they were in small enclosures housed in pairs, and then when they moved into larger enclosures housed in social groups. Focussing on two movement behaviours that are commonly studied in animal personality research (daily distance travelled and residence time)^10,16^ we investigated several aspects of calf behavioural variation. Daily distance travelled was chosen as a measure of activity as it reflects how much an animal moves each day. Residence time is defined as the time an individual spends inside a circle centred around its location, without leaving it for more than a specified amount of time. This indicates how much time an animal spends investigating each area before moving on and therefore is indicative of exploratory behaviour^10,16^. We expected to see consistent variation between individuals in movement behaviours that persisted across time and different environmental contexts^17,28,29^ (prediction 1) representing personality differences. However, we also expected to see plasticity^10,16^ and anticipated that individuals would move larger distances (prediction 2) and spend less time in one place (prediction 3) in the social housing compared to the pair housing since they have increased space available to them^30^. We also expected to see a link between personality and plasticity^8^ and predicted that those individuals that moved the largest distances and stayed still the least in the pair housing would change their behaviour the most (increase distances, reduce time stationary) in the social housing (prediction 4) since exploratory phenotypes are often observed to show greater plasticity in response to environmental changes^8^. Finally, because of the close proximity of individuals in the pair housing, we expected social conformity effects^23,31–33^ where pen-mates would show greater similarity in distance travelled and residence times than other randomly chosen individuals in the population (prediction 5). Lastly, we investigated the potential for carry-over effects from the pair housing^34–36^, where the behaviour in the group housing may be affected by the individuals previous experience in the pair housing, and specifically partner personality (prediction 6).

## Materials and methods

### Ethical approval

The study was approved by the Ethics Committee of the School of Veterinary Medicine and Science, University of Nottingham (unique reference number 1481150603). All methods were performed in accordance with the relevant guidelines and regulations and are reported in accordance with the ARRIVE guidelines^37^.

### Animals, housing and farm management

**Figure 1:**
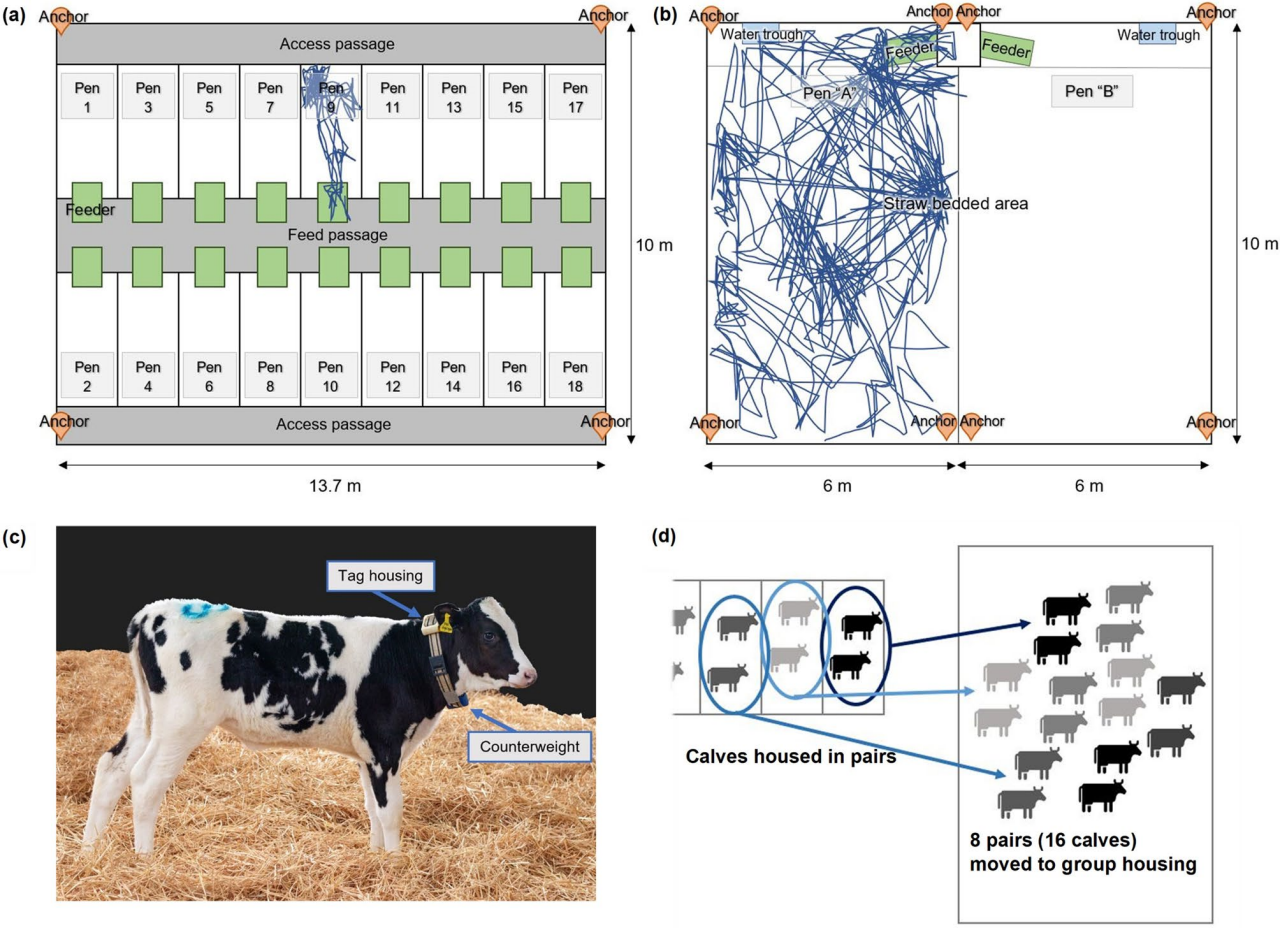
Diagrams of the pair housing (**a**) and group housing (**b**) indicating the position of the location anchors, and an example of the trajectory of one calf for 1 day for each of the housings which is obtained from positional data collected by collars worn by all calves (**c**). Calves are moved with their pen-partner into social groups of 16 individuals (**d**).

The study took place at the Centre for Dairy Science Innovation at the University of Nottingham, UK, between April 2021 and July 2022. A total of 90 Holstein Friesian dairy heifer calves were divided into 6 cohorts of up to 16 calves as per normal farm management (Table 1), enrolled between April 2021 and January 2022. The accepted standard for studies measuring repeatability and intercept-slope correlations is a minimum sample size of 20 individuals^38^, so this study fulfils these requirements, and a minimum of 6 cohorts was required in order to model cohort as a random effect. The calves were first housed in pairs in straw bedded 1.5 m × 3.5 m pens from birth, as per regular farm management (Fig. 1a). Each pen had an automatic feeder which dispensed milk replacer to the calves and had troughs for concentrates, chopped straw and water. Each calf had an individual daily allowance of milk replacer which started from 6 L at 2 days of age and increased gradually until it reached 10 L at 40 days of age. A cohort of up to 16 calves was formed by the eight pairs of calves closest in age. When the youngest pair was at least 2 weeks old the calves were moved to one of the two adjacent straw-bedded 6 m × 10 m pens in their cohort group of 16 (Fig. 1b,d) where they stayed for 9–10 weeks. There the calves were fed a daily allowance of 10 L of milk replacer from the same type of automatic feeder and had *ad-lib* access to concentrates, chopped straw and water. The weaning process started with a gradual decrease of the allowance after 37 days from the move to the group pen and completed after 57 days. For a more detailed description of the feeding protocols and allowances see Carslake et al.^39^.

**Table 1:** We studied six cohorts of N = 16 calves, tracking the majority or all individuals in each cohort.

Cohort (N = 16)	Calves tracked	Calves not tracked	Age of tracked calves
1	11	5	35.6
2	16	0	29.6
3	16	0	39.7
4	16	0	37.6
5	15	1	33.0
6	16	0	41.5
Total	90	6	36.6

Five calves in the first cohort were excluded: one due to severe illness in the pair housing, another due to being a bull calf and three more due to being Holstein Friesian and Aberdeen Angus crosses (i.e., different breeds). One calf was excluded from the fifth cohort due to a sensor malfunction leading to data loss. Age (mean at day 0 in group housing) for tracked calves is also provided.

### Sensor data collection

Calves wore Ultra-Wideband Sewio Leonardo iMU tags, (Noldus, Wageningen, the Netherlands)^21^ housed in counter-weighted collars (Fig. 1c), which logged the relative local coordinates (x,y) of individuals via triangulation using anchors (Fig. 1a,b) at 1 Hz. Collars were fitted at the earliest opportunity from when the calves were at least 4 days old. Calves were monitored from minimum 7 days old in the pair housing in order to exclude the first days of life, when they were often handled to train calves to use the automatic feeder. The monitoring continued in the pair housing until the youngest individual in the cohort was at least 2 weeks old (mean number of monitored days: 20.8 days, range 4–38 days). In the group housing calves were monitored for the first 20 days that they were in the group pen to have a comparable number of days in both housings.

Tags were validated in each housing location by placing tags at multiple static positions. We assessed the precision of the location data by calculating the mean circular error probability (CEP). CEP represents the radius of a circle where 50% of the location points reported by the location tag lie, and centred at the mean location^40^. Accuracy was calculated as the mean distance between the ground truth location and the location reported by the sensor (DIST)^40^. In the pair housing the CEP and DIST were 0.11 [0.06–0.17] m and 0.12 [0.05–0.16] m respectively, while in the group housing they were 0.15 [0.12–0.28] m and 0.17 [0.13–0.33] m.

### Pre-processing and cleaning of positional data

When people or vehicles moved in or around the housing, these time periods were removed from the dataset. This amounted to 9045 h of the total 85,320 calf-hours (representing 10.6% of all data). If any location points fell outside the pen coordinates these were also removed from the data. The position data was smoothed using a moving average over a 10-s window and then this smoothed data was used to compute movement parameters.

### Movement parameters

The daily distance travelled was computed as the sum of the distance between consecutive points over 24 h for each day using the smoothed coordinates reported by the tags. Residence time is a measure of how much time an individual spends stationary, and we used the time spent inside a circle of a 1-m radius centred around its location (nearly ten times our location error), without leaving it for more than 1 min, and the time was averaged over 24 h. These two behaviours were chosen because they are among the most frequently used in studies that detect personality types from location and movement data^10,16,17^.

### Statistical analysis

Statistical analysis was carried out using R software v4.2.2^41^. We used mixed effect linear models to investigate personality and plasticity in daily distance travelled and residence time using the lme4 package in R^42^, as:

1 $$Y_{ijk} = \left( {\beta_{0} + \, coh_{0k} + \, ind_{0jk} } \right) + \, \beta_{1} X_{1ijk} + \cdots + \, \beta_{4} X_{4ijk} + \, e_{0ijk} .$$

In Eq. (1) *Y*_*ijk*_ is the movement metric (distance travelled or residence time) for each event (*i*), for individual (*j*)*,* in cohort (*k*). *β*_0_ is the mean value of individual responses, *coh*_0*k*_ is the random component of the intercept representing the cohort effect and *ind*_0*jk*_ represents the individual contribution^43^. *β*_1_–*β*_4_ represent the vector coefficients for each of the fixed effects (*X*_1_–*X*_4_): day of observation (continuous), calf age (on the first day in the group pen), housing (pair, group), and health of the calf (“healthy”, “sick” or “convalescent”, determined based on the treatment records as describe in Occhiuto et al.^17^). The random intercept (*V*_*ind*0_) represents the variance between individuals and is assumed to be normally distributed (*N*) with zero mean and variance (*Ω*_*ind*_). The residual error *e*_*0ij*_ represents the variance within individuals (*V*_*eo*_) and is assumed to be normally distributed with zero mean and variance (*Ω*_*e*_)^43^.

Personality (adjusted repeatability, *R*) for each movement behaviour was calculated by extracting the variation due to the random effect (*V*_*ind0*_) and dividing it by the total phenotypic variation (*V*_*ind*0_ + *V*_*eo*_)^16,43–46^:

2 $${\text{R}}\text{=}\frac{\text{Vind0}}{\text{(V}{\text{ind0}}+ \text{ V} \text{eo)}}.$$

Plasticity was determined as the change in behaviour across housing type. To determine if there was a correlation between the personality and plasticity, we ran the model above adding the housing as a random slope:

3 $$Y_{ijk} = \left( {\beta_{0} + \, coh_{0k} + \, ind_{0jk} } \right) + \, \left( {\beta_{{1}} + ind_{1jk} } \right)X_{1ijk} + \cdots + \, \beta_{4} X_{4ijk} + \, e_{0ijk} .$$

The Pearson correlation coefficient between the personality and plasticity was computed from the intercept and slope extracted from the model.

To test whether calves that were pen-mates were more similar to each other than any other pair of calves, we ran a GLMM for each behaviour and each housing and extracted the intercept for each calf. We then compared the coefficient of variation between the differences in the intercepts of the two pen-mates to the coefficient of variation of the difference in the intercept between pairs of calves obtained by randomly permuting calf IDs, and calculated the proportion of times that the coefficient of variation of the pen-mates was equal or less than the one for the randomly paired individuals. The test was performed for both behaviours in the pair and group housing using 10,000 permutations.

To test whether the behaviour of the pen-mates in the pair housing had any effect on the behaviour of the calves in the group housing we extracted the intercept for each calf and their pen-mate for the two movement behaviours in the pair housing and added them as fixed effects into two new GLMMs with other fixed and random effects retained as described above.

﻿﻿﻿﻿The analysis was run once including instances of disease in the calves and once excluding them. As the results were very similar, we have reported the results of the analysis which included and controlled for disease. The results with healthy calves only can be found in the supplementary material﻿. 

## Results

We measured repeatability across two housings and predicted consistent variation between individuals in movement behaviours (prediction 1) representing personality differences. We also predicted behavioural plasticity in the distance travelled and residence time between the two housings (prediction 2 and 3) and a link between personality and plasticity by measuring the intercept-slope correlation of random slope mixed effects linear models (prediction 4). Additionally, we predicted greater similarity among pen-mates in their movement (prediction 5) and potential effects of partner personality (prediction 6).

### Personality

**Figure 2:**
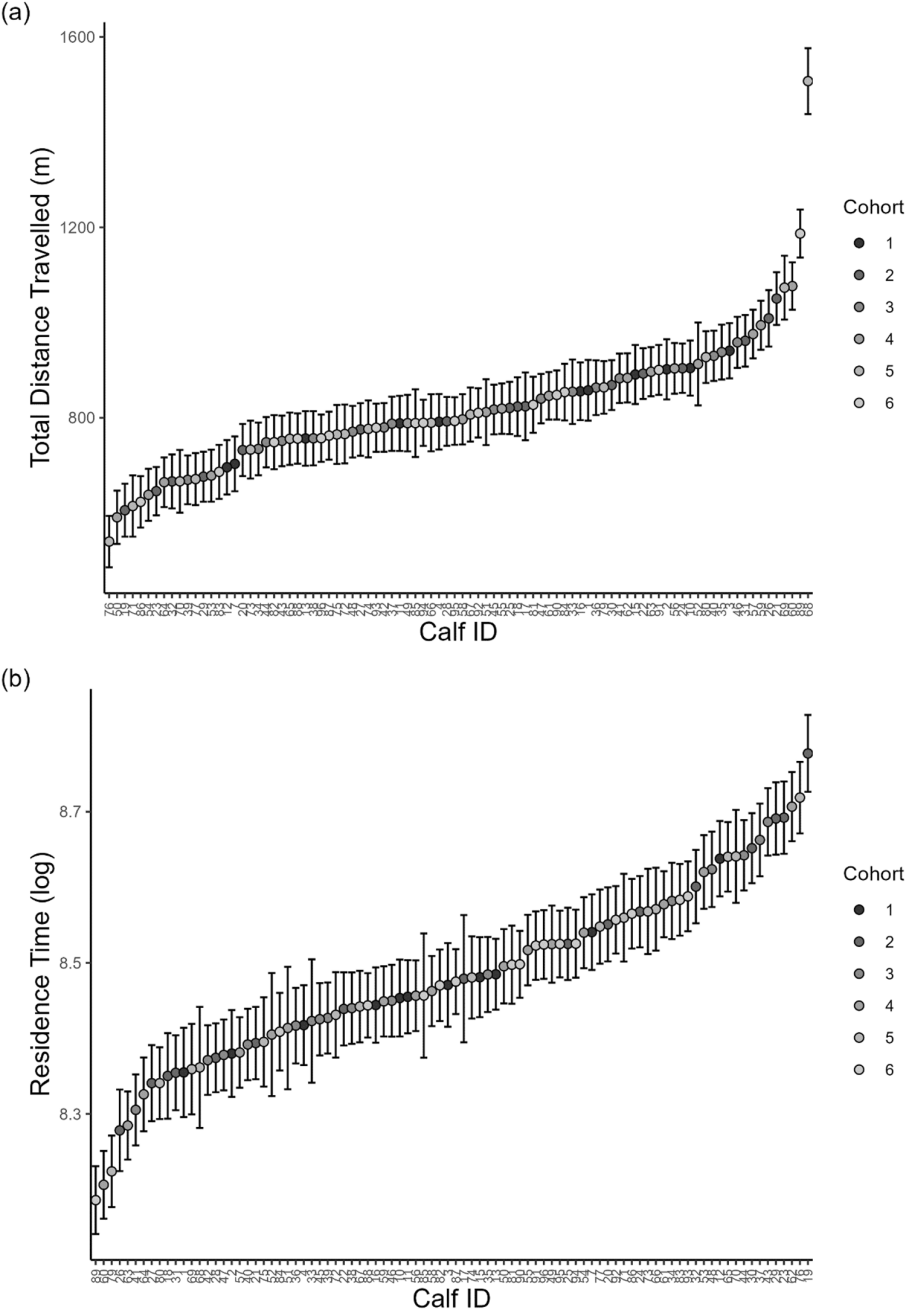
Calf (n = 90) individual distribution of (**a**) distance travelled and (**b**) residence time for each individual. Estimates are derived from mixed effect linear models after controlling for day, age of the calf (on the first day in the group pen), housing and health status. The error bars represent the standard deviation.

Distance travelled (Fig. 2a) and residence time (Fig. 2b) were repeatable over time and across contexts (housing types), representative of personality traits (prediction 1). The repeatability for distance travelled was R = 0.25 ([0.22, 0.28], N = 90) and for residence time was R = 0.22 ([0.19, 0.24], N = 90). The effect sizes and parameters of the fixed effects and random effects of the models are available in the supplementary material (Table S1).

### Plasticity

**Figure 3:**
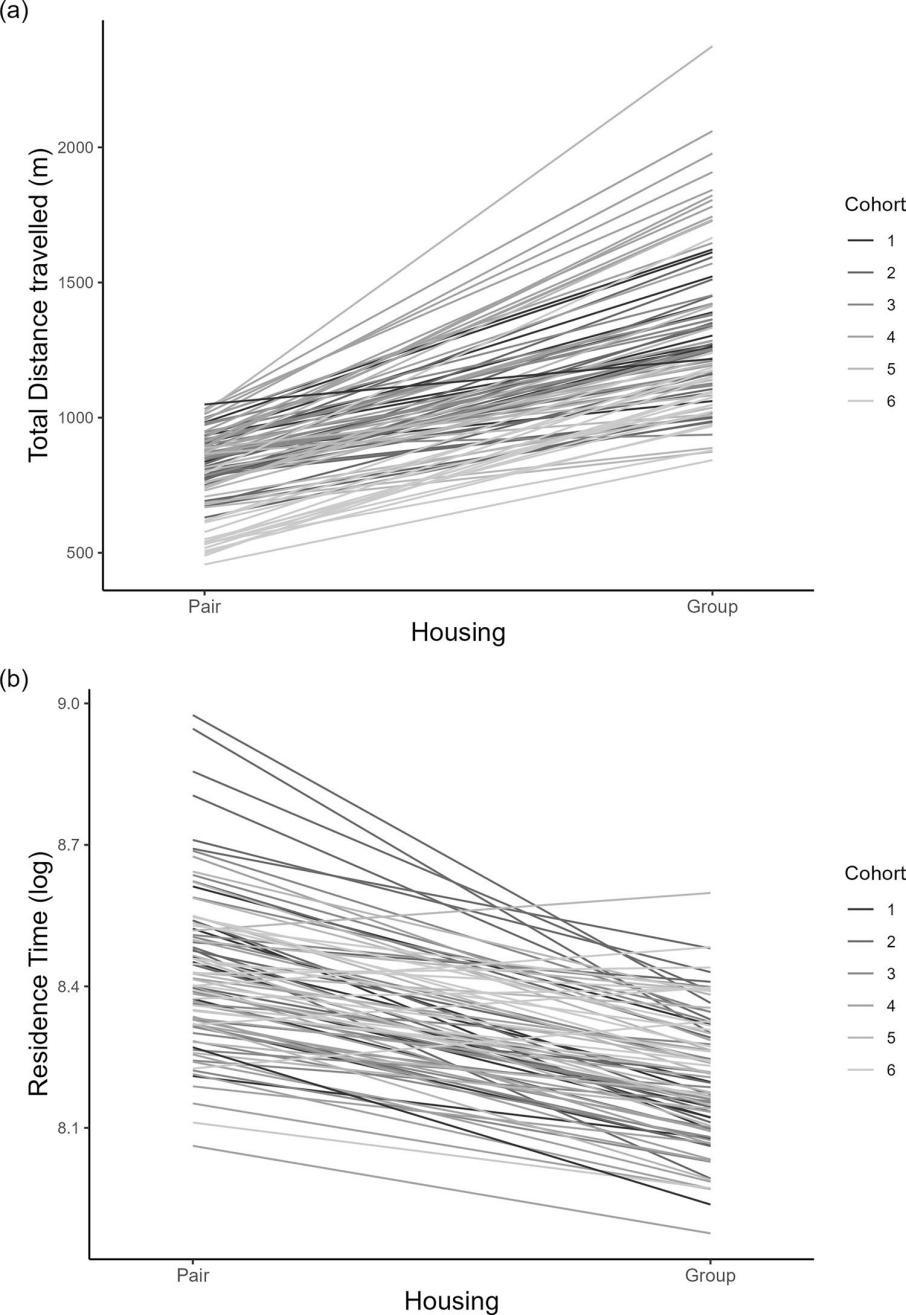
Intercept (personality) and slope (plasticity) for distance travelled (**a**) and residence time (**b**) for each individual. Estimates are derived from mixed effect linear models after controlling for day, age of the calf (on the first day in the group pen), housing and health status.

Individuals travelled further (distance travelled: β = 496.2, SE = 9.8, p < 0.001; Fig. 3a), and spent less time in one location (residence time: β =  − 0.22, SE = 0.01, p < 0.001; Fig. 3b) showing plasticity in behaviour and supporting predictions 2 and 3. The effect sizes and parameters of the fixed effects and random effects of the models are available in the Supplementary Material (Table S2).

### Personality-plasticity correlation

Individual personality (the intercept from our models) predicted plasticity (the slope from our models) for distance travelled and residence time, supporting prediction 4. The correlation was positive for distance travelled (cor = 0.57, p < 0.001) and negative for residence time (cor = − 0.65, p < 0.001), meaning that the calves which moved furthest and spent more time in one location showed the greatest changes when moved to the social housing (Fig. 3a,b).

### Pen-mate effects

Calves that were pen-mates in the pair housing were significantly more similar to each other in their distance travelled (test statistic = 147.7, df = 1806, p < 0.001) and residence time (test statistic = 21,194.74, df = 1806, p < 0.001) while in the pair housing, supporting prediction 5, but this similarity did not carry-over to the group housing (distance travelled: test statistic = 1171.45, df = 1806, p = 0.44; residence time: test statistic = 3578.41, df = 1806, p = 0.14) contrary to prediction 6. The mean distance travelled by the pen-mate in the pair housing had a significant negative effect on the distance travelled by the calves in the group housing (β = − 0.79, SE = 0.30, p = 0.01), but there was no significant effect for the residence time (β = − 0.03, SE = 0.10, p = 0.73).

## Discussion

This study is the first to measure personality and plasticity in dairy calf movement behaviours across different contexts. We established that movement behaviours were repeatable across both time and contexts supporting the existing evidence of movement-based personality traits in cattle. Our results also showed that calf personality predicted the change in movement across contexts, providing evidence for personality-dependent behavioural plasticity.

The daily distance travelled by the calves and the residence time were repeatable for both housings, indicating that individuals were consistently different from each other in these behaviours. This agrees with previous evidence of personality traits from movement measures in calves^17^, and adds evidence of the contextual stability of these traits. The repeatability of distance travelled can be interpreted as an “active” personality trait, which drives how much individuals move around the available space despite considerable changes in pen size and structure across the different housings^17,18,47^. Residence time is a measure of how long an individual stays in one location before moving again and can therefore be interpreted as a measure of “exploratory” tendency^10,16^. Consistent differences in residence time again persisted with the changes in housing, indicating that this is also an inherent characteristic of the individuals. Consistent differences in activity and exploration in farmed animals—as measured by movement behaviours—mirrors work conducted in-situ with wild animals such as brown bears, African elephants and chacma baboons^10,16,47^.

The overall increase in distance travelled from the pair to the group housing can be explained by an increase in available space as the calves went from a pen measuring just over 5 m^2^ (2.63 m^2^ per calf) to one measuring 60 m^2^ (3.75 m^2^ per calf). In addition to reduction in stocking density, the group housing has much longer distances between the areas of interest for the calves (feeder, water trough, food trough and lying space), which is likely to cause more walking. While this alone could have resulted in an incentive to walk longer distances daily, the move to the second housing was also concurrent with the mixing of eight pairs of calves into one pen. It is likely that mixing with new individuals could have also contributed to an increase in distance travelled as the calves might be moving around the pen to interact with each other^48^. The decrease in residence time from the pair to the group housing can similarly be explained by the change in space available to move; it is less likely that an individual will stay in the same location when there is more space available to move in, and more frequent/different social interactions may cause more frequent movement^49^. It is therefore likely that both changes in the physical and social environment caused predictable changes in behaviour, and further studies that manipulate the environmental conditions are needed to disentangle the effects of space availability and social dynamics.

The correlation between the intercept and slope for distance travelled and residence time indicates the existence of personality-dependent behavioural plasticity. This means that individuals that were at the higher end of the spectrum for distance travelled increased more when moved to the group housing than did the individuals that were on the lower end. The same was true for residence time where there was an overall decrease between the housings but the individuals that were at the higher end of the spectrum had a sharper decrease. Calves were therefore affected differently by the change in environment depending on their personality type. It is likely that the calves with a more active personality were more severely constrained in their behaviour when in the small area of the pair housing and were able to express their preferred behavioural levels in a larger pen, while the less active individuals did not increase as much. Again, our findings are similar to studies in wild populations that showed personality dependent changes in movement behaviours when individuals are moving in different environments. For example, a study of baboons moving in both natural and urban spaces in Cape Town, South Africa found that individuals that travelled straighter paths on average, travelled even straighter paths in urban spaces^10^. This highlights the importance of considering the environmental component and behavioural plasticity when assessing animal personality as different settings might yield different results^7^. Here, measuring movement behaviours only in the pair housing or only in the group housing would have given different results for individuals’ mean and variability in behaviour.

The similarity between pen-mates for both behaviours is likely a result of social conformity effects, where individuals change their behaviour to match others^23,31–33^. However, this similarity did not persist in the group housing, indicating no “carry-over” effect of the original pairs’ behaviour. This is somewhat unexpected since a previous study where calves were managed in the same way reported that individuals that had been pen-mates had more social interactions with those individuals in the group housing^48^. Therefore, it might be that calves express their inherent personality in the larger space/social context whilst maintaining association preferences for their former pen-mate. Evidence from both adult cattle and calves demonstrated individual differences in the level of sociality^50,51^. This means that individuals may have been affected differently by their pen-mate and therefore including a measure of sociality may be of interest for future studies. Additionally, being paired with a more active pen-mate (with a higher mean of distance travelled in the pair housing) was associated with a decreased distance travelled in the group housing, which supports the interpretation of an inherent personality trait. Specifically, whilst we find individuals show social conformity in the pair housing, after moving to the group housing they are “released” from this constraint and express their personality type. Another possibility is that this change is a result of increased number and type of social interactions, causing individuals becoming more similar (i.e., conform to synchronise with the group) or divergent (i.e., differentiate to exploit different niches) from the average behaviour of the whole group they are in^33^. The fact that individuals are clustered by cohort (darker/lighter lines are close to each other in Fig. 3) suggests some evidence for the former, and again, will be a fruitful area for further work.

Overall, the results of this study highlight the importance of considering the physical and social context that the behaviour of farm animals is measured in, as it can greatly impact the extent of personality differences between individuals. Our finding that dairy calves have consistent personality traits, which also affect how they respond to changes in housings, could be used to inform farm animal research and practices related to health and welfare^20^, since individuals might have different requirements or susceptibilities to stress and disease^52–54^. Future research should therefore investigate the links between personality, plasticity and health or productivity to determine the impact of these differences. These results also highlight the potential for using the high-resolution movement data collected by precision livestock technology, in conjunction with statistical models, to quantify behavioural variation between and within individuals. Finally, we provided evidence for the presence of social conformity effects on calf personality, indicating that the personality of the individuals needs to be considered when forming social pairings or groups. Further studies are needed to understand how personality traits are shaped in young animals and what factors could affect their development.

### Supplementary Information

Supplementary Information.

## Data Availability

Data to reproduce the results in this study is available from the corresponding author upon reasonable request.
