# Publisher Correction: Evidence of personality-dependent plasticity in dairy calf movement behaviours derived from automated data collection

**DOI:** 10.1038/s41598-023-47360-w

**Published:** 2023-11-16

**Authors:** Francesca Occhiuto, Jorge A. Vázquez-Diosdado, Andrew J. King, Jasmeet Kaler

**Affiliations:** 1 School of Veterinary Medicine and Science, University of Nottingham, Sutton Bonington Campus, Leicestershire, LE12 5RD UK; 2 Department of Biosciences, Faculty of Science and Engineering, Singleton Park Campus, Swansea University, Swansea, SA2 8PP UK

Correction to: *Scientific Reports* 10.1038/s41598-023-44957-z, published online 25 October 2023

The original HTML version of this Article contained a display error in Equation 2.

$$R=\frac{Vind0}{(V\{ind0\} + V\{eo)\}}$$

Equation 2 now reads:

$$R=\frac{Vind0}{(Vind0 + Veo)}$$

This error has now been corrected in the HTML version of the Article; the PDF version was correct from the time of publication.

