# Supplementary material for: Evidence of personality-dependent plasticity in dairy calf movement behaviours derived from automated data collection
Source: Sci Rep. 2023 Oct 25;13:18243. doi: 10.1038/s41598-023-44957-z (PMC10600154; doi:10.1038/s41598-023-44957-z)
Supplement: Supplementary file 1 — Supplementary Information. [file 41598_2023_44957_MOESM1_ESM.docx]

**Evidence of personality-dependent plasticity in dairy calf movement behaviours derived from automated data collection**

Francesca Occhiuto^1^*, Jorge A. Vázquez-Diosdado^1^, Andrew J. King^2^ and Jasmeet Kaler^1^*

^1^School of Veterinary Medicine and Science, University of Nottingham, Sutton Bonington Campus, Leicestershire, LE12 5RD, UK

^2^Department of Biosciences, Faculty of Science and Engineering, Singleton Park Campus, Swansea University, Swansea, SA2 8PP, UK

[*francesca.occhiuto@nottingham.ac.uk](mailto:*francesca.occhiuto@nottingham.ac.uk)

[*jasmeet.kaler@nottingham.ac.uk](mailto:*jasmeet.kaler@nottingham.ac.uk)

**Table S1:** Effect sizes and parameters of the fixed effects and random effects of the random intercept linear models for each variable of interest: total walked distance and residence time.

|  | | **Distance travelled** | | | | **Residence time (log-transformed)** | | | |
| --- | --- | --- | --- | --- | --- | --- | --- | --- | --- |
| **Fixed effects** | | | | | | | | | |
|  | Observations | Estimate | St.Error | | p-value | Estimate | St.Error | | p-value |
| (Intercept) |  | 818.19 | 98.25 | | <0.001 | 8.48 | 0.07 | | <0.001 |
| Day | 3555 | -2.79 | 0.59 | | <0.001 | 0.001 | 0.001 | | 0.10 |
| Age | 3555 | 1.57 | 2.03 | | 0.44 | -0.002 | 0.002 | | 0.17 |
| Health status - Healthy | 2681 | Ref. | - | | - | Ref. | - | | - |
| Health status - Convalescent | 353 | -11.77 | 15.24 | | 0.44 | -0.02 | 0.01 | | 0.16 |
| Health status - Sick | 521 | -139.36 | 13.39 | | <0.001 | 0.11 | 0.01 | | <0.001 |
| Housing - Group | 1678 | 496.23 | 9.85 | | <0.001 | -0.22 | 0.01 | | <0.001 |
| **Random effects** | | | | | | | | | |
| **Groups** | N | Variance | | St.Dev | | Variance | | St.Dev | |
| Calf (intercept) | 90 | 21562.00 | | 146.80 | | 0.02 | | 0.13 | |
| Cohort (intercept) | 6 | 24226.00 | | 155.60 | | 0.003 | | 0.06 | |
| Residual | - | 66852.00 | | 258.60 | | 0.06 | | 0.25 | |
| Marginal R2 | | 0.37 | | | | 0.15 | | | |
| Conditional R2 | | 0.63 | | | | 0.36 | | | |

**Table S2:** Effect sizes and parameters of the fixed effects and random effects of the random slope linear models for each variable of interest: total walked distance and residence time.

|  | | **Distance travelled** | | | | **Residence time (log-transformed)** | | | |
| --- | --- | --- | --- | --- | --- | --- | --- | --- | --- |
| **Fixed effects** | | | | | | | | | |
|  | Observations | Estimate | St.Error | | p-value | Estimate | St.Error | | p-value |
| (Intercept) |  | 826.29 | 82.17 | | <0.001 | 8.48 | 0.07 | | <0.001 |
| Day | 3555 | -3.81 | 0.58 | | <0.001 | 0.001 | 0.001 | | 0.13 |
| Age | 3555 | 2.05 | 1.30 | | 0.12 | -0.002 | 0.002 | | 0.21 |
| Health status - Healthy | 2681 | Ref. | - | | - | Ref. | - | | - |
| Health status - Convalescent | 353 | 15.05 | 13.99 | | 0.28 | -0.02 | 0.01 | | 0.29 |
| Health status - Sick | 521 | -126.92 | 12.66 | | <0.001 | 0.11 | 0.01 | | <0.001 |
| Housing - Group | 1678 | 480.41 | 26.54 | | <0.001 | -0.22 | 0.02 | | <0.001 |
| **Random effects** | | | | | | | | | |
| **Groups** | N | Variance | | St.Dev | | Variance | | St.Dev | |
| Calf (intercept) | 90 | 6401.00 | | 80.01 | | 0.03 | | 0.16 | |
| Calf (Housing - Group) | 90 | 55484.00 | | 235.55 | | 0.02 | | 0.15 | |
| Cohort (intercept) | 6 | 25127.00 | | 158.52 | | 0.004 | | 0.06 | |
| Residual | - | 53411.00 | | 231.11 | | 0.06 | | 0.24 | |
| Marginal R2 | | 0.35 | | | | 0.15 | | | |
| Conditional R2 | | 0.71 | | | | 0.42 | | | |

**Results from the analysis excluding sick and convalescent calves.**

Distance travelled (Figure S1a) and residence time (Figure S1b) were repeatable over time and across contexts (housing types), representative of personality traits (prediction 1). The repeatability for distance travelled was R=0.26 ([0.23, 0.30], N=90) and for residence time was R= 0.24 ([0.21, 0.27], N=90). The effect sizes and parameters of the fixed effects and random effects of the models are summarised in table S3.


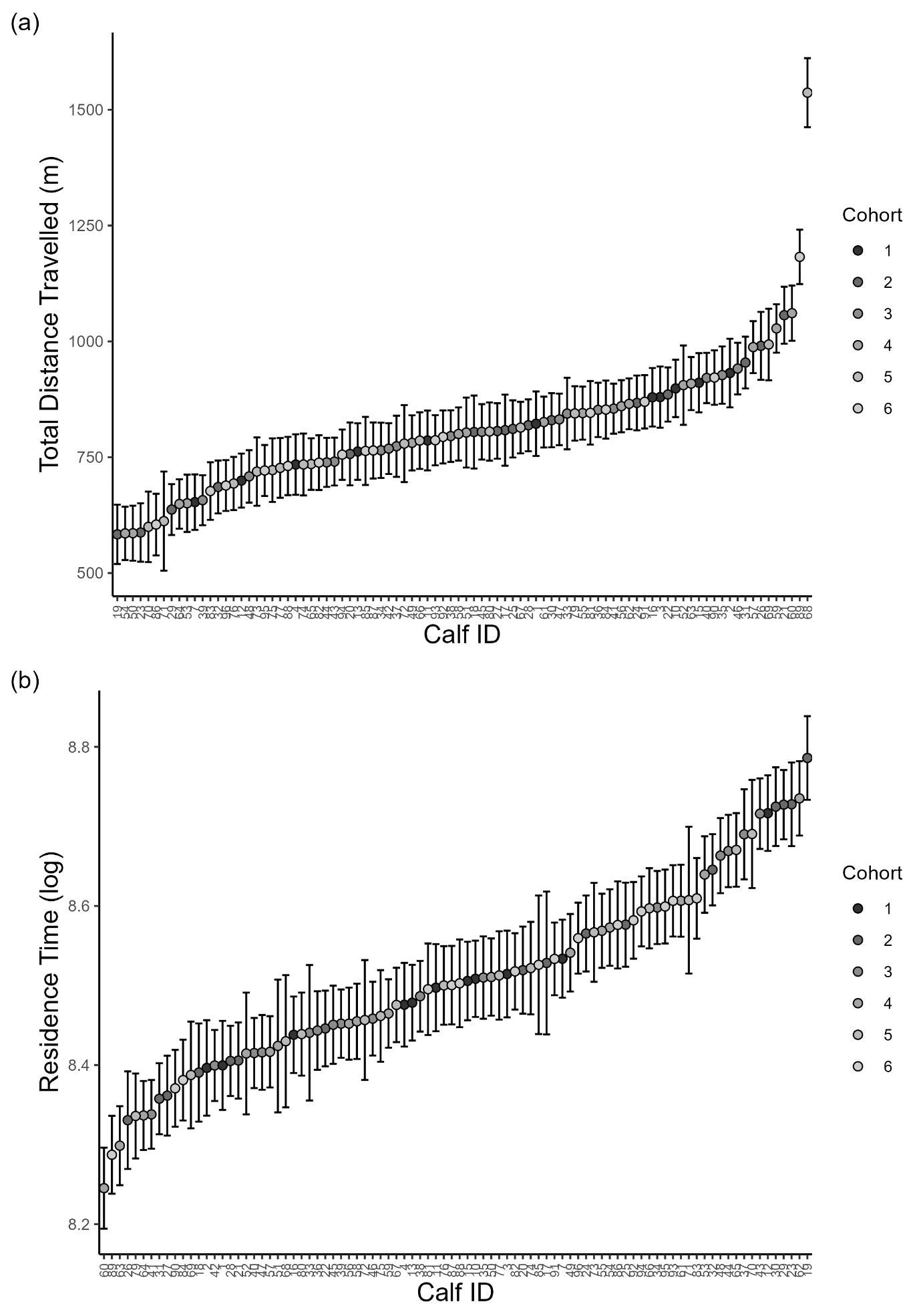


**Figure S1:** Calf (n=90) individual distribution of (a) distance travelled and (b) residence time for each individual excluding any times when they were classified as sick or convalescent. Estimates are derived from mixed effect linear models after controlling for day, age of the calf (on the first day in the group pen), housing and health status. The error bars represent the standard deviation.

**Table S3:** Effect sizes and parameters of the fixed effects and random effects of the random intercept linear models including healthy calves only for each variable of interest: total walked distance and residence time.

|  | | **Distance travelled** | | | | **Residence time (log-transformed)** | | | |
| --- | --- | --- | --- | --- | --- | --- | --- | --- | --- |
| **Fixed effects** | | | | | | | | | |
|  | Observations | Estimate | St.Error | | p-value | Estimate | St.Error | | p-value |
| (Intercept) |  | 808.15 | 99.62 | | <0.001 | 8.51 | 0.07 | | <0.001 |
| Day | 2681 | -2.35 | 0.70 | | <0.001 | <0.001 | <0.001 | | 0.99 |
| Age | 2681 | 1.50 | 2.12 | | 0.48 | -0.002 | 0.002 | | 0.14 |
| Housing - Group | 1214 | 509.41 | 11.73 | | <0.001 | -0.24 | 0.01 | | <0.001 |
| **Random effects** | | | | | | | | | |
| **Groups** | N | Variance | | St.Dev | | Variance | | St.Dev | |
| Calf (intercept) | 90 | 21562.00 | | 146.80 | | 0.02 | | 0.13 | |
| Cohort (intercept) | 6 | 24226.00 | | 155.60 | | 0.002 | | 0.05 | |
| Residual | - | 66852.00 | | 258.60 | | 0.05 | | 0.23 | |
| Marginal R2 | | 0.37 | | | | 0.16 | | | |
| Conditional R2 | | 0.63 | | | | 0.38 | | | |
